# Erwinia carotovora Quorum Sensing System Regulates Host-Specific Virulence Factors and Development Delay in Drosophila melanogaster

**DOI:** 10.1128/mBio.01292-20

**Published:** 2020-06-23

**Authors:** Filipe J. D. Vieira, Pol Nadal-Jimenez, Luis Teixeira, Karina B. Xavier

**Affiliations:** a Instituto Gulbenkian de Ciência, Oeiras, Portugal; b Faculdade de Medicina da Universidade de Lisboa, Lisbon, Portugal; Michigan State University; University of Hawaii at Manoa

**Keywords:** *Drosophila*, *Ecc15*, homoserine lactones, bacterial infections, host-pathogen interactions, insect development, invertebrate-microbe interactions, quorum sensing

## Abstract

Multihost bacteria have to rapidly adapt to drastic environmental changes, relying on a fine integration of multiple stimuli for an optimal genetic response. Erwinia carotovora spp. are phytopathogens that cause soft-rot disease. Strain *Ecc15* in particular is a model for bacterial oral-route infection in Drosophila melanogaster as it harbors a unique gene, *evf*, that encodes the *Erwinia* virulence factor (Evf), which is a major determinant for infection of the D. melanogaster gut. However, the factors involved in the regulation of *evf* expression are poorly understood. We investigated whether *evf* could be controlled by quorum sensing as, in the *Erwinia* genus, quorum sensing regulates pectolytic enzymes, the major virulence factors needed to infect plants. Here, we show that transcription of *evf* is positively regulated by quorum sensing in *Ecc15* via acyl-homoserine lactone (AHL) signal synthase ExpI and AHL receptors ExpR1 and ExpR2. We also show that the load of *Ecc15* in the gut depends upon the quorum sensing-mediated regulation of *evf*. Furthermore, we demonstrate that larvae infected with *Ecc15* suffer a developmental delay as a direct consequence of the regulation of *evf* via quorum sensing. Finally, we demonstrate that *evf* is coexpressed with plant cell wall-degrading enzymes (PCWDE) during plant infection in a quorum sensing-dependent manner. Overall, our results show that *Ecc15* relies on quorum sensing to control production of both pectolytic enzymes and Evf. This regulation influences the interaction of *Ecc15* with its two known hosts, indicating that quorum sensing signaling may impact bacterial dissemination via insect vectors that feed on rotting plants.

## Abstract (precis)

Integration of genetic networks allows bacteria to rapidly adapt to changing environments. This is particularly important in bacteria that interact with multiple hosts. Erwinia carotovora is a plant pathogen that uses Drosophila melanogaster as a vector. To interact with these two hosts, *Ecc15* uses different sets of virulence factors: plant cell wall-degrading enzymes to infect plants and the *Erwinia* virulence factor (*evf*) to infect *Drosophila*. Our work shows that, despite the virulence factors being specific for each host, both sets are coactivated by homoserine lactone quorum sensing and by the two-component GacS/A system in infected plants. This regulation is essential for *Ecc15* loads in the gut of *Drosophila* and minimizes the developmental delay caused by the bacteria with respect to the insect vector. Our findings provide evidence that coactivation of the host-specific factors in the plant may function as a predictive mechanism to maximize the probability of transit of the bacteria between hosts.

## INTRODUCTION

Insects play an important role in the dissemination of microorganisms that cause both human and plant diseases. This dissemination may be an active process whereby microbes develop strategies to interact with insects and use them as vectors (1, 2). To do so, bacteria must have the ability to persist within the host (either lifelong or transiently), evading or resisting its immune system in order to abrogate their elimination (3, 4). The host vector responds with a battery of innate defenses, such as production of antimicrobial peptides and reactive oxygen species as well as behavioral strategies (e.g., avoidance) and physiological responses (e.g., increased peristalsis) (5–9). The successful establishment of these interactions, from the bacterial perspective, ultimately depends on maximizing the fitness of the microorganism and minimizing the impact on the fitness of the vector host (1). Phytopathogenic bacteria such as *Phytoplasma* spp., Xylella fastidiosa, Pantoea stewartii (formerly Erwinia stewartii), and Erwinia carotovora (also known as Pectobacterium carotovorum) are among those known to establish close associations with insects and to rely on these hosts as vectors, presumably to facilitate rapid dissemination among plants (10–13). Thus, understanding the molecular mechanisms governing the establishment of these interactions is crucial to prevent insect-borne diseases.

Bacteria from the *Erwinia* genus produce pectolytic enzymes that degrade plant tissue, causing soft root disease (14). These bacteria survive poorly in soil, overwinter in decaying plant material (14), and use insects, including *Drosophila* species (12, 15) as vectors. Specifically, the nonlethal interaction between the phytopathogen Erwinia carotovora (strain *Ecc15*) and Drosophila melanogaster has been used as a model to study bacterium-host interactions. Oral infections with *Ecc15* lead to a transient systemic induction of the immune system in D. melanogaster and consequent production of antimicrobial peptides (7, 16). During infection, *Ecc15* causes damage and loss of epithelial cells, leading to an overall shrinkage of the gut (7). To reestablish the normal functions of the gut, there is activation of tissue repair programs with proliferation and differentiation of stem cells, which have been correlated with a developmental delay in the larval stage of *Drosophila* (7, 17). These responses are strain specific and highly dependent on the expression levels of the *Erwinia* virulence factor gene (*evf*) (18), which promotes bacterial infection of the *Drosophila* gut by an unknown mechanism (19). Additionally, expression of *evf* requires the transcriptional regulator Hor (18), but the signals required for the activation of this regulator remain unknown.

**FIG 1:**
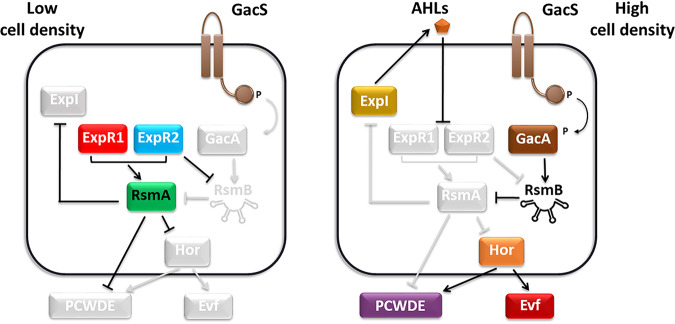
Signaling pathways regulating PCWDE and *evf* production in *Erwinia* spp. At low cell density, when the concentration of AHL signaling molecules is low, ExpR1 and ExpR2 induce transcription of *rsmA*, repressing expression of both PCWDE and *evf*. As cell density increases, AHLs accumulate and when the concentration threshold is reached these signal molecules bind to ExpR1 and ExpR2 receptors, inhibiting their DNA binding ability. As a result, *rsmA* transcription is no longer induced. The GacS/A two-component system is also active at high cell density and promotes transcription of *rsmB*, a noncoding RNA that has high binding affinity to RsmA and inhibits the remaining available RsmA. Inhibition of RsmA results in increased production of both PCWDE and *hor* and, consequently, *evf*, leading to full induction of virulence. We show here that *evf* is regulated by quorum sensing and the GacS/A system via *hor*. While *evf* is not necessary to infect the plant host, we also show that there is coexpression of *evf* and PCWDE during plant infection. Gray and black lines indicate inactive and active pathways, respectively. Arrows indicate activation, while intersecting lines indicate repression.

Quorum sensing has recently been shown to be important in the regulation of bacterial traits that affect the persistence and/or virulence of bacteria in insects (20–23). Many bacteria use quorum sensing to regulate gene expression as a function of population density (24, 25). This cell-cell signaling mechanism relies on the production, secretion, and response to extracellular signaling molecules called autoinducers (25–27). Bacteria from the *Erwinia* genus produce a mixture of plant cell wall-degrading enzymes (PCWDE), which are the major virulence factors used to degrade plant tissues and potentiate bacterial invasion of the plant host (28–31). In these bacteria, expression of these PCWDE is tightly regulated by two main signaling pathways: the acyl-homoserine lactone (AHL) quorum sensing system and the GacS/A two-component (GAC) system (Fig. 1) (32–36). Typically, the AHL quorum sensing system present in *Erwinia* spp. includes the AHL synthase ExpI (37) and two AHL receptors, ExpR1 and ExpR2 (38), which are homologues of the canonical LuxI/R quorum sensing system first identified in Vibrio fischeri (39–41). The GacS/A two-component system is also activated at high cell density, and, like the AHL quorum sensing system, regulates virulence in many Gram-negative pathogenic bacteria (42–47). Given the importance of these two signal transduction pathways for the expression of the major plant virulence factors in *Erwinia* spp., we investigated whether quorum sensing and the GacS/A system also regulate *evf* expression in *Ecc15*. Additionally, we tested whether these signaling pathways are important for *Ecc15* infection and determined the consequences of this interaction for the insect host. Our results show that PCWDE expression and *evf* expression in *Ecc15*, which are required for the interactions with plants and insects, respectively, are regulated by the same quorum sensing signaling pathway. Moreover, we demonstrate that the quorum sensing-dependent *evf* expression has a negative effect on the insect host as it leads to a developmental delay in larvae infected with *Ecc15*. Finally, we show that *evf* and the PCWDE are coexpressed during the plant infection, which may function as a predictive mechanism to maximize the probability of transmission of *Erwinia* to a new host.

## RESULTS

### Expression of *evf* is regulated by both AHL-dependent quorum sensing and the GAC system.

**FIG 2:**
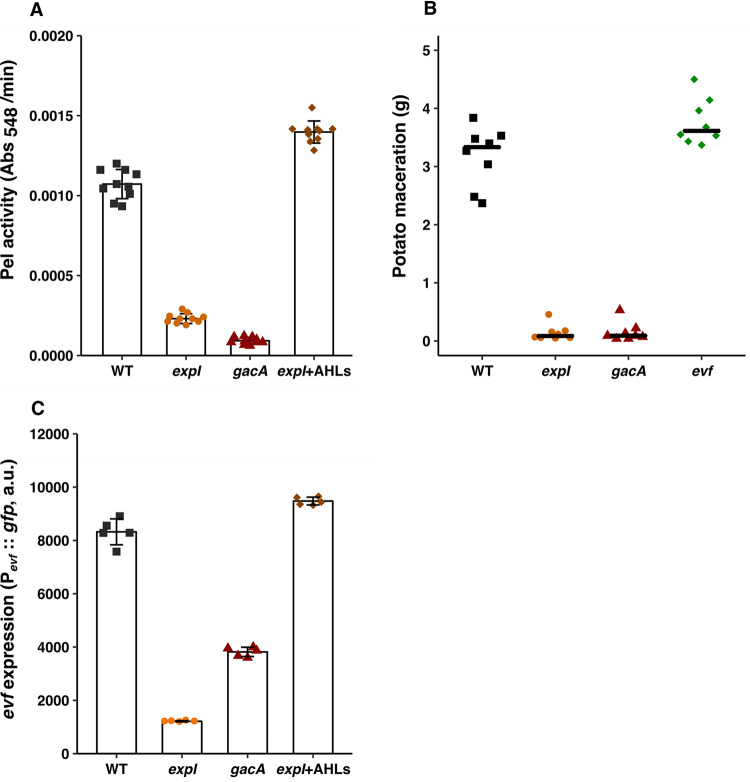
Production of pectate lyase and expression of *evf* are dependent on both quorum sensing and the GAC system. (A) Pectate lyase activity in cell-free supernatants of WT *Ecc15* and *expI* and *gacA* mutants at 6 h of growth in LB plus 0.4% PGA, *n* = 10. (B) Potato maceration quantification (grams) in potatoes infected with WT *Ecc15* and *expI*, *gacA*, and *evf* mutants, 48 h postinfection, *n* = 8. (C) Pevf::*gfp* expression in WT *Ecc15*, *expI* and *gacA* mutants, and *expI*+AHLs at 6 h of growth in LB + Spec, *n* = 5. Complementation with AHLs (*expI*+AHLs) was performed with a mixture of 1 μM 3-oxo-C6-HSL and 3-oxo-C8-HSL. Growth curves of the strains used are shown in Fig. S1. Error bars represent standard deviations of the means. For each panel, results from a representative experiment from three independent experiments are shown (the results from the other two experiments are shown in Fig. S2). Results of statistical analysis taking the data of all three experiments are shown in Fig. S2. a.u., arbitrary units.

We first investigated whether activation of the production of PCWDE in *Ecc15* requires both the AHL quorum sensing system and the GacS/A two-component (GAC) system, as occurs in other members of the *Erwinia* (or *Pectobacterium*) genus (33, 35, 37, 48). We constructed deletion mutants of *expI* and *gacA*, the genes encoding homologues of the AHL synthase and the response regulator of the GAC system, respectively. We determined whether any of these mutations cause a growth defect in *Ecc15*, and observed no difference in growth compared to the wild-type (WT) strain (see Fig. S1 in the supplemental material). We then measured pectate lyase (Pel) activity in supernatants of cultures from the *Ecc15* WT strain or the *expI* or *gacA* mutants, as this is one of the PCWDE typically secreted by *Erwinia* spp. As shown in Fig. 2A (and for replicate experiments in Fig. S2), both the *expI* and the *gacA* mutants exhibited pronounced reductions in pectate lyase activity compared to the WT strain (Tukey honestly significant difference [HSD] test, *P < *0.001; see also Fig. S2C). Addition of a mixture of exogenous 3-oxo-C6-HSL and 3-oxo-C8-HSL, the major AHLs produced by Erwinia carotovora (48), to an *expI* mutant culture was sufficient to restore production of this PCWDE to levels higher than those seen with the WT (Fig. 2A, Tukey HSD test, *P < *0.001; see also Fig. S2C). In addition, both the *expI and gacA* mutants were impaired in virulence for the plant host, which we tested by measuring the mass of macerated tissue in potato tubers inoculated with these genotypes (Fig. 2B, Tukey HSD test, *P < *0.001; see also Fig. S2F). In contrast, the *evf* mutant showed no statistically significant difference in maceration in comparison to the WT (Fig. 2B; see also Fig. S2D to F). Altogether, these results show that production of pectate lyase and plant host virulence are regulated by both the AHL and GAC systems in *Ecc15*, as occurs in other *Erwinia* spp., where *expI* and *gacA* mutants have been shown to be avirulent (35, 36, 49, 50). Moreover, we show that *evf* is not necessary for plant infection (Fig. 2B; see also Fig. S2D to F).

10.1128/mBio.01292-20.1 FIG S1 Growth curves of WT *Ecc15*, *expI, gacA*, and *expI* + AHL mutants carrying a P*_evf_*::*gfp* reporter fusion. Download FIG S1, TIF file, 0.1 MB. Copyright © 2020 Vieira et al. 2020 Vieira et al. This content is distributed under the terms of the Creative Commons Attribution 4.0 International license.

10.1128/mBio.01292-20.2 FIG S2 Independent replicates of the experiments described in the Fig. 2 legend (production of pectate lyase and expression of *evf* are dependent on both quorum sensing and the GAC system). (A and B) Replicates of experiments described in the Fig. 2A legend. (C) Statistical groups of results of all three experiments described in the Fig. 2A legend. (D and E) Replicates of experiments described in the Fig. 2B legend. (F) Statistical groups representing all three experiments described in the Fig. 2B legend. (G and H) Replicates of experiments described in the Fig. 2C legend. (I) Statistical groups representing all three experiments described in the Fig. 2C legend. Statistical analysis was performed using a linear mixed-effect model. A Tukey HSD test was applied for multiple comparisons using the estimates obtained from the model. Download FIG S2, TIF file, 0.9 MB. Copyright © 2020 Vieira et al. 2020 Vieira et al. This content is distributed under the terms of the Creative Commons Attribution 4.0 International license.

To investigate whether *evf* expression is also regulated by these two systems, we analyzed the expression of a transcriptional reporter consisting of a green fluorescent protein (GFP) fused to the promoter of *evf* (P*_evf_*::*gfp*) in mutants of either AHL quorum sensing or GAC signaling systems. We observed that the expression of P*_evf_*::*gfp* was reduced in the *expI* mutant compared to the WT (Tukey HSD test, *P < *0.001) and that this expression was restored when exogenous AHLs were supplied to the culture (Fig. 2C; see also Fig. S2G to I). In the *gacA* mutant, expression of the *evf* promoter was also reduced compared to the WT, but not as much as in the *expI* mutant (Fig. 2C, Tukey HSD test, *P < *0.001; see also Fig. S2G to I). Since it was previously shown that mutants in the GAC system produce lower levels of AHLs (35, 51), we asked if the difference observed between the WT and the *gacA* mutant could be explained solely by the lower levels of AHLs produced by the latter. However, addition of exogenous AHLs to the cultures of a *gacA* mutant did not restore the levels of P*_evf_*::*gfp* expression to WT levels (Fig. S3). Therefore, we conclude that the *gacA* phenotype regarding *evf* expression is mostly independent of AHLs. Moreover, complementation of the *gacA* mutant with a *gacA* gene in *trans* restored the levels of *evf* expression (Fig. S4). Overall, these results show that full activation of both *evf* expression and PCWDE activity is dependent on quorum sensing regulation via AHLs and, to a lesser extent, on activation of the GAC system.

10.1128/mBio.01292-20.3 FIG S3 AHLs cannot complement intermediate levels of *evf* expression in a *gacA* mutant. Data represent P*_evf_*::*gfp* expression in WT *Ecc15* and the *gacA* mutant at 6 h of growth in LB + Spec, *n* = 3. Complementation with AHLs was performed with a mixture of 1 μM 3-oxo-C6-HSL and 3-oxo-C8-HSL. Error bars represent standard deviations of the means. Download FIG S3, TIF file, 0.1 MB. Copyright © 2020 Vieira et al. 2020 Vieira et al. This content is distributed under the terms of the Creative Commons Attribution 4.0 International license.

10.1128/mBio.01292-20.4 FIG S4 Complementation of *gacA*, *expR1*, and *expR2* genes. Data represent *evf* expression at 6 h of growth in WT *Ecc15* and in *expI*, *expI expR1 expR2*, and *gacA* mutants (white bars) or in the *gacA* mutant complemented with the *gacA* gene in *trans* (light gray) and the *expI expR1 expR2* mutant complemented with *expR1* and *expR2* genes in *trans* (dark gray), *n* = 5. “Vector” stands for pOM1 P*_evf_*::*gfp*, while “p(*gacA*^+^)” and “p(*expR1^+^ expR2^+^*)” refer to the same plasmid expressing *gacA* and *expR1 expR2*, respectively. Error bars represent standard deviations of the means. Download FIG S4, TIF file, 0.2 MB. Copyright © 2020 Vieira et al. 2020 Vieira et al. This content is distributed under the terms of the Creative Commons Attribution 4.0 International license.

**FIG 3:**
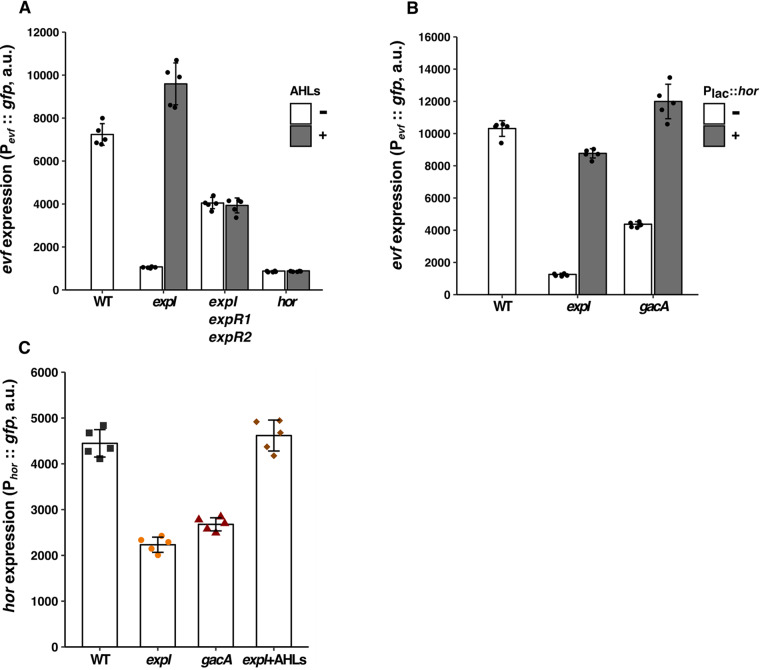
*evf* regulation by quorum sensing is dependent on ExpR receptors and *hor*. (A) P*_evf_*::*gfp* expression without (white bars) or with (gray bars) addition of exogenous AHLs in the *Ecc15* WT and in *expI*, *expI expR1 expR2*, and *hor* mutants at 6 h of growth in LB + Spec, *n* = 5. (B) P*_evf_*::*gfp* expression in the *Ecc15* WT strain and *expI* and *gacA* mutants containing a plasmid with the P*_evf_*::*gfp* fusion (white bars) or with both P*_lac_*::*hor* and P*_evf_*::*gfp* fusions (gray bars) at 6 h of growth in LB + Spec, *n* = 5. (C) P*_hor_*::*gfp* expression in WT *Ecc15* and *expI* and *gacA* mutants at 6 h of growth in LB + Spec, *n* = 5. Complementation with AHLs was performed with a mixture of 1 μM 3-oxo-C6-HSL and 3-oxo-C8-HSL. Error bars represent standard deviations of the means. For each panel, results from a representative experiment from three independent experiments are shown (results from the other two experiments are shown in Fig. S5). Results of statistical analysis taking the data of all three experiments are shown in Fig. S5. a.u., arbitrary units.

In the absence of AHLs, the AHL receptors ExpR1 and ExpR2 were found to be associated with repression of virulence traits such as PCWDE (Fig. 1) (37, 52). These receptors are DNA binding proteins that act as transcriptional activators of *rsmA*, which encodes a global repressor of quorum sensing-regulated genes in *Erwinia* spp. (38, 52, 53). Upon AHL binding, these receptors lose their ability to bind DNA, resulting in decreased expression of *rsmA* and, consequently, increased expression of virulence traits (Fig. 1) (54, 55). To determine whether ExpR1 and ExpR2 also mediate AHL-dependent regulation of *evf* expression, we constructed deletions of these two genes in the *expI* background. We measured expression of the P*_evf_*::*gfp* reporter in this *expI expR1 expR2* triple mutant, with or without exogenous AHLs. Because AHLs block activation of RsmA via ExpR1 and ExpR2 (54, 55), deletion of *expR1 and expR2* in the *expI* background was expected to result in the derepression of *evf*. Consistent with this prediction, P*_evf_*::*gfp* expression was higher in the *expI expR1 expR2* triple mutant than in the *expI* single mutant (Fig. 3A, Tukey HSD test, *P < *0.001; see also Fig. S5A to C) and the levels of *evf* expression in an *expI expR1 expR2* mutant complemented with the *expR1* and *expR2* genes in *trans* were similar to those seen with the *expI* mutant (Fig. S4). However, the expression levels of P*_evf_*::*gfp* were lower in the *expI expR1 expR2* mutant than in the WT (Fig. 3A, Tukey HSD test, *P < *0.001; see also Fig. S5A to C). The fact that deletion of these two receptors in the *expI* background was not sufficient to fully restore expression of *evf* to WT levels indicates that additional regulators control the expression of *evf*. Nonetheless, while addition of exogenous AHLs to a culture of an *expI* mutant increased P_evf_::*gfp* expression, it remained unaltered in the triple *expI expR1 expR2* mutant (Fig. 3A, Tukey HSD test, *P = *1; see also Fig. S5A to C). Therefore, AHL-dependent regulation of *evf* expression is mediated by *expR1* and *expR2*, as is also the case for the regulation of PCWDE in other *Erwinia* spp. (34, 36, 49).

10.1128/mBio.01292-20.5 FIG S5 Independent replicates of the experiment shown in Fig. 3a (*evf* regulation by quorum sensing is dependent on ExpR receptors and *hor*). (A and B) Replicates of experiments described in the Fig. 3A legend. (C) Statistical groups representing all three experiments described in the Fig. 3A legend. (D and E) Replicates of experiments described in the Fig. 3B legend. (F) Statistical groups representing all three experiments described in the Fig. 3B legend. (G and H) Replicates of experiments described in the Fig. 3C legend. (I) Statistical groups representing all three experiments described in the Fig. 3C. Statistical analysis was performed using a linear mixed-effect model. A Tukey HSD test was applied for multiple comparisons using the estimates obtained from the model. Download FIG S5, TIF file, 0.9 MB. Copyright © 2020 Vieira et al. 2020 Vieira et al. This content is distributed under the terms of the Creative Commons Attribution 4.0 International license.

### Regulation of *evf* by AHL quorum sensing is mediated by *hor*.

It was previously shown that Hor, a global regulator of diverse physiological processes in many animal-pathogenic and plant-pathogenic bacterial species (56), is a positive regulator of *evf* (18) and that, as in other *Erwinia* spp., *hor* is regulated by quorum sensing (57). Therefore, we asked if AHL-dependent regulation of *evf* is mediated via *hor*. We analyzed the expression levels of the P*_evf_*::*gfp* reporter in a *hor* mutant and found that the levels were lower than in the WT and as low as those in the *expI* mutant (Fig. 3A). Moreover, we observed that addition of exogenous AHLs to a *hor* mutant did not restore the expression of *evf* (Fig. 3A, Tukey HSD test, *P = *1; see also Fig. S5A to C). We next cloned the *hor* gene under the control of a *lac* promoter in the plasmid containing the P*_evf_*::*gfp* fusion and measured *evf* expression levels in the *expI* and *gacA* mutants expressing or not the *hor* gene. We observed that expression of *hor* in either the *expI* or the *gacA* mutant restored *evf* expression to levels similar to those seen with the WT (Fig. 3B, Tukey HSD test, *P < *0.001; see also Fig. S5D to F). Therefore, regulation of *evf* is mediated by both the AHL and the GAC systems and occurs via *hor*. Next, we asked whether these systems regulate *hor* itself by analyzing the expression of a *hor* promoter fusion (P*_hor_*::*gfp*) in *expI* and *gacA* mutants. As with the *evf* reporter, we observed that P*_hor_*::*gfp* expression was lower in an *expI* mutant than in the WT strain (Fig. 3C, Tukey HSD test, *P < *0.001; see also Fig. S5G to I). Moreover, expression of P*_hor_*::*gfp* was complemented to WT levels by the addition of exogenous AHLs to the growth medium of the *expI* mutant (Fig. 3C, Tukey HSD test, *P = *0.08; see also Fig. S5G to I). These data demonstrate that *hor* expression is regulated by AHLs and is necessary for the increase of *evf* expression mediated by AHLs.

### Infection by *Ecc15* causes a developmental delay in D. melanogaster larvae in a manner dependent on quorum sensing and GAC regulation of *evf* expression.

**FIG 4:**
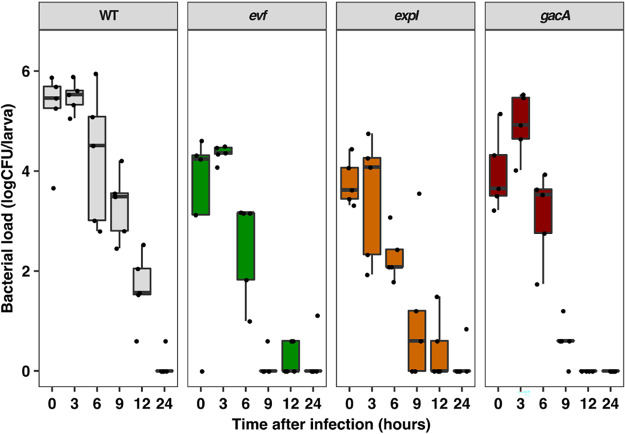
*Ecc15* loads are higher in D. melanogaster larvae orally infected with the WT than in those infected with mutants impaired in *evf* expression. D. melanogaster L3 stage larvae were infected with WT *Ecc15* and *evf*, *expI*, and *gacA* mutants for 30 min and then transferred to fresh media. Following the infection period, CFU levels of *Ecc15* were measured at the specified time points. Each dot represents CFU of one single larvae (5 larvae per time point). The time point indicated as 0 h after infection corresponds to 30 min of confined exposure to 200 μl of an OD_600_ of 200. Results from a representative experiment from three independent experiments are shown (results from the other two experiments are shown in Fig. S5). Results of statistical analysis of the comparisons of data from the entire infection period for each condition tested in all three experiments are shown in Fig. S6.

It is known that Evf promotes infection in the D. melanogaster gut (18, 19). To examine the effects of downregulation of *evf* on quorum sensing and GAC mutants, we measured *Ecc15* loads upon oral infection. We inoculated *Ecc15* WT or *evf*, *expI*, or *gacA* mutant cells into D. melanogaster L3 stage larvae and assessed the dynamics of bacterial loads by counting the number of CFU of *Ecc15* over time. As previously reported (19), *Ecc15* infection was transient and larvae were able to clear it after 24 h (Fig. 4). Additionally, we observed that the rates of elimination of the bacteria from the larval gut were not statistically significantly different between the WT and the *evf*, *gacA*, and *expI* mutants (Fig. 4, linear mixed model [lmm], chi-square test *P = *0.27). However, we also observed that *Ecc15* WT loads were approximately 10 times higher than the *evf* mutant loads considering the entire infection period (Fig. 4, Tukey HSD test, *P < *0.001; see also Fig. S6), confirming that *evf* is required for optimal infection of the larval gut by *Ecc15*. Importantly, a similar trend was observed in comparisons of the WT to the *gacA* and *expI* mutants, the two mutants impaired in *evf* expression (Fig. 4, Tukey HSD test, *P < *0.001; see also Fig. S6), revealing the importance of quorum sensing regulation and the GAC system in the infection process. Taken together, our data show that *evf* provides *Ecc15* with the ability to reach high loads in the insect gut but does not increase its capacity to survive inside it.

10.1128/mBio.01292-20.6 FIG S6 Independent replicates of the experiment shown in Fig. 4 (*Ecc15* loads are higher in D. melanogaster larvae orally infected with WT than in mutants impaired in *evf* expression). (A and B) Replicates of experiments described in the Fig. 4 legend. (C) Statistical groups representing all three experiments described in the Fig. 4 legend. Statistical analysis was performed using a linear mixed-effect model. A Tukey HSD test was applied for multiple comparisons using the estimates obtained from the model. Download FIG S6, TIF file, 0.3 MB. Copyright © 2020 Vieira et al. 2020 Vieira et al. This content is distributed under the terms of the Creative Commons Attribution 4.0 International license.

**FIG 5:**
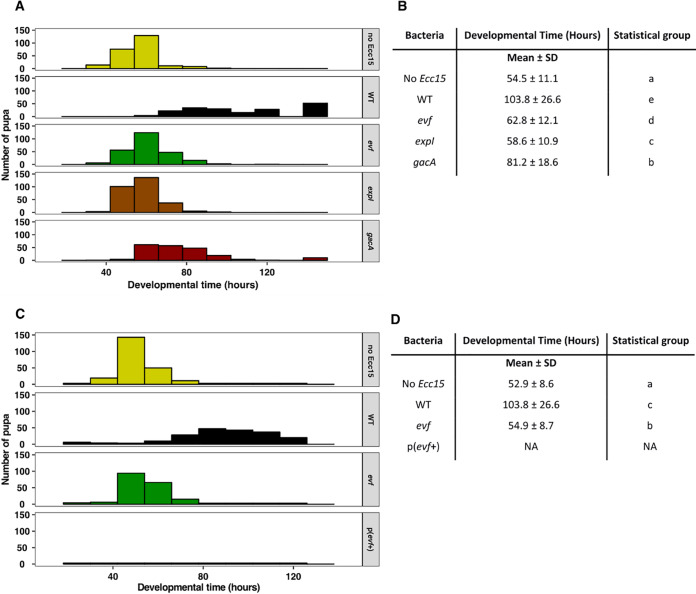
*Ecc15* causes a developmental delay in D. melanogaster larvae that is dependent on *evf*, quorum sensing, and the GAC system. (A and C) L3 stage *Drosophila* larvae pupariation time after exposure to (A) WT *Ecc15* and *evf*, *expI*, and *gacA* mutants or (C) WT *Ecc15* overexpressing Evf [p(*evf*^+^)], compared with noninfected larvae. (B and D) Average developmental time (in hours) with standard deviation (data correspond to the results shown in panels A and C, respectively). Overexpression of Evf was lethal, as larva exposed to WT *Ecc15* overexpressing Evf (C) died without reaching the pupa stage. NA, not applicable. Results from a representative experiment of three independent experiments are shown (results from the other two experiments are shown in Fig. S7). The statistical groups represented in panels B and D were determined using a linear mixed-effect model taking into consideration the data from the three experiments. A Tukey HSD test was applied for multiple comparisons using the estimates obtained from the model.

Next, we asked if infection of D. melanogaster larvae by *Ecc15* has an effect on larval development given that larvae infected with *Ecc15* are shorter than noninfected larvae (58). To investigate this possibility, we infected D. melanogaster L3 stage larvae orally with *Ecc15* WT or an *evf* mutant and followed their development over time. We found that infection by WT *Ecc15* delayed passage of D. melanogaster larvae to the pupal stage an average of 49 h, compared to noninfected larvae (Fig. 5A and B; see also Fig. S7; Tukey HSD test, *P < *0.001). Moreover, we show that this strong delay was *evf* dependent, since larvae exposed to an *evf* mutant showed a delay of only 8 h compared to noninfected larvae (Tukey HSD test, *P < *0.001, Fig. 5A and B). We then asked if the mutants in the quorum sensing pathway and GAC system, which have low expression of *evf*, would show a similar phenotype. We observed that larvae exposed to the *expI* mutant, which has very low expression of *evf*, also show a delay of only 4 h with respect to noninfected larvae, similarly to the *evf* mutant (Tukey HSD test, *P < *0.001, Fig. 5A and B). Interestingly, larvae infected with the *gacA* mutant, which has intermediate levels of *evf* expression, showed an intermediate developmental delay, taking an average of 27 h longer than noninfected larvae to reach the pupal stage (Tukey HSD test, *P < *0.001, Fig. 5A and B). Since the developmental delay correlated with the levels of *evf* expression in the strains tested, we next examined whether constitutive overexpression of *evf* would exacerbate the phenotype. We observed that larvae infected with a WT *Ecc15* overexpressing *evf* died before reaching the pupal stage (Fig. 5C and D). These results show that *Ecc15* has a negative impact on larval development and that this effect requires both *evf* and the quorum sensing and GAC regulatory systems.

10.1128/mBio.01292-20.7 FIG S7 Independent replicates of the experiment shown in Fig. 5 (*Ecc15* causes a developmental delay in D. melanogaster larvae that is dependent on *evf*, quorum sensing, and the GAC system). (A and B) Replicates of experiments described in the Fig. 5A legend. (C and D) Replicates of experiments described in the Fig. 5C. Larvae exposed to WT *Ecc15* overexpressing Evf died without reaching the pupa stage. Download FIG S7, TIF file, 0.3 MB. Copyright © 2020 Vieira et al. 2020 Vieira et al. This content is distributed under the terms of the Creative Commons Attribution 4.0 International license.

### *evf* and *pelA* are coexpressed during plant infection.

**FIG 6:**
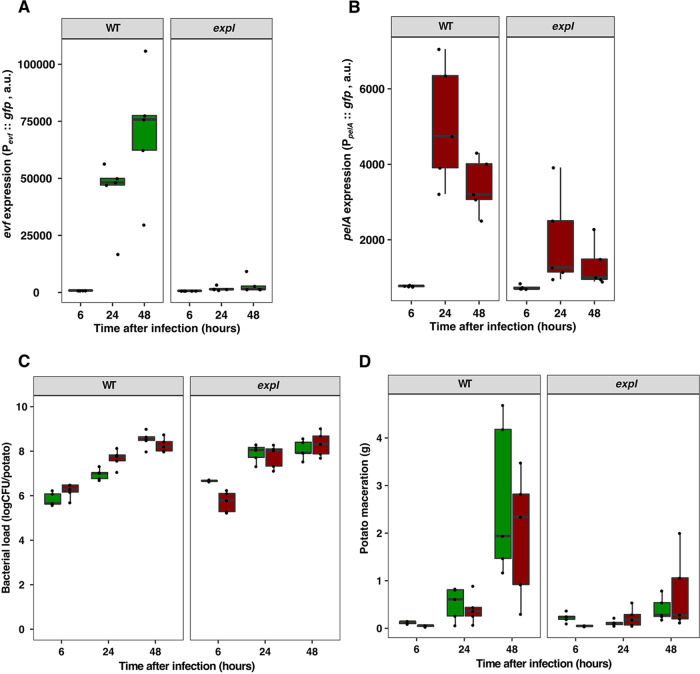
Coactivation of *evf* and *pelA* expression in plant infections. Potatoes were infected for 6, 24, or 48 h with a WT *Ecc15* strain or an *expI* mutant carrying an *evf* or *pelA gfp* reporter plasmid with a constitutive mCherry promoter. At each specified time point, *pelA* or *evf* expression, CFU levels, and weight of macerated plant tissue were determined. (A and B) P*_evf_*::*gfp* expression (A) and P*_pelA_*::*gfp* expression (B) in potatoes infected with WT *Ecc15* or *expI* mutant. (C) CFU of WT *Ecc15* or *expI* mutant expressing mCherry and carrying the P*_evf_*::*gfp* (green boxes) or the P*_pelA_*::*gfp* (red boxes) reporter plasmids. (D) Potato maceration (in grams) in potatoes infected with WT *Ecc15* or *expI* mutant. Each dot represents an independent potato, *n* = 5. Results are from a representative experiment of two independent experiments (results from the second experiment are shown in Fig. S8). Results of statistical analysis of comparisons of data from the entire infection period for each condition tested using the data from both experiments are shown in Fig. S8.

Considering that the interaction of *Ecc15* with both insects and plants is thought to be crucial for the life cycle of this bacterial species and that quorum sensing is essential for the expression of both *evf* and PCWDE, we asked if *evf* expression is coactivated with *pelA* (one of the PCWDE) in a quorum sensing-dependent manner during plant infection. We observed that in WT *Ecc15*, both *evf* and *pelA* promoter fusions were expressed 24 h postinfection of potato tubers (Fig. 6A and B). Moreover, we observed that the levels of both *evf* expression and *pelA* expression were higher in the WT *Ecc15* strain than in an *expI* mutant (*t* test, *P < *0.01, Fig. 6A and B; see also Fig. S8), even though the bacterial loads at the site of infection were similar for the WT and *expI* strains (*t* test, *P = *0.37, Fig. 6C; see also Fig. S8). As expected, the levels of plant tissue maceration were higher in potatoes infected with *Ecc15* WT than in potatoes infected with the *expI* mutant (*t* test, *P < *0.01, Fig. 6D; see also Fig. S8). Overall, our results show that there is quorum sensing-dependent coactivation of *evf* and *pelA* during plant infection as well as plant tissue maceration.

10.1128/mBio.01292-20.8 FIG S8 Independent replicate of the experiment described in the Fig. 6 legend. (*evf* expression and *pelA* expression are coactivated during plant infection). (A) Replicate of experiment shown in Fig. 6A legend. (B) Replicate of experiment shown in Fig. 6B legend. (C) Replicate of experiment shown in Fig. 6C legend. (D) Replicate of experiment shown in Fig. 6D legend. (E) Statistical groups representing the two experiments described in the Fig. 6 legend. Statistical analysis was performed using a linear mixed-effect model. A *t* test was applied using the estimates from the model. Download FIG S8, TIF file, 0.7 MB. Copyright © 2020 Vieira et al. 2020 Vieira et al. This content is distributed under the terms of the Creative Commons Attribution 4.0 International license.

## DISCUSSION

*Erwinia* spp. are phytopathogenic bacteria thought to depend on insects to spread among plant hosts (1, 12, 13). To interact with both plants and insects, *Ecc15* relies on different traits that seem to be specific for the interaction with each host. In this bacterium, PCWDE are the major virulence factors required for plant infection (42) and Evf is required to infect D. melanogaster but is not necessary to infect potato tubers (Fig. 2B) (16, 18). It was not known whether WT *Ecc15*, which relies on multiple hosts for survival, regulates host-specific traits using the same or different signal transduction networks. Here, we showed that the AHL-dependent ExpI/ExpR system, which regulates plant virulence factors (Fig. 1), is also essential for the expression of the insect virulence factor *evf*, suggesting that the signal transduction networks regulating traits required across hosts are the same. An *expI* mutant had lower levels of *evf* expression than the WT, and WT levels could be restored by addition of exogenous AHLs to the growth medium. We also demonstrated that the GAC system, which is thought to respond to the physiological state of the cell (44) and is involved in regulation of plant virulence factors (43, 59), is also necessary for full expression of *evf*. Additionally, we showed that regulation by these two networks occurs through *hor*, a conserved transcriptional regulator of the SlyA family (60), previously found to be regulated by quorum sensing in another E. carotovora strain (57). ExpR1 and ExpR2 AHL receptors function as activators of *rsmA*, the global repressor of the AHL regulon; therefore, we expected the *expI expR1 expR2* mutant to have the same levels of *evf* expression as the *expI* mutant supplemented with AHLs. However, we found that the *expI expR1 expR2* mutant has lower levels of *evf* expression than both the *expI* mutant supplemented with AHLs and the WT. Moreover, we showed that complementation of the *expI expR1 expR2* mutant with AHLs does not change the level of *evf* expression. These results show that *expR1* and *expR2* are required for the response of *Ecc15* to AHLs but also indicate that an additional AHL-independent regulator is playing a role in the regulation of *evf* in this bacterium. One possibility is that *Ecc15* has additional orphan *luxR* genes encoding a DNA binding protein homologous to LuxR that lack a cognate AHL synthase. These orphan genes are divided into two categories: those that have both a LuxR DNA and an AHL binding domain, such as ExpR2, and those that have only the typical LuxR DNA binding domain (61), such as *vqsR* in Pseudomonas aeruginosa. In this bacterium, in response to an unknown signal, *vqsR* has been found to downregulate expression of virulence through binding to the promoter region of the quorum sensing receptor *qscR*, inhibiting its expression without responding to AHLs (62). Because addition of exogenous AHLs to the *expI expR1 expR2* mutant does not change the level of *evf* expression, this unknown regulator is more likely to lie within the second category of orphan LuxR receptors. Our data also suggest that this unknown regulator could be repressed by *rsmA*, since the *expI* mutant shows lower levels of *evf* expression than the *expI expR1 expR2* mutant. Another layer of regulation required for PCWDE expression in *Erwinia* spp. is represented by the detection of external environmental signals such as pectin, a component of the plant cell wall (63, 64). In the absence of plant signals, transcription of PCWDE is repressed. Unlike in the regulation of PCWDE in *Erwinia* spp., we have no evidence for the need of a host signal in our experimental setting for *evf*, since we were able to detect *evf* expression in cells grown in LB without the need for other signals. However, this does not exclude the possibility that environmental signals, perhaps related to insect-derived compounds, have a role in the overall levels of *evf* expression.

It has been hypothesized that *evf* was horizontally acquired by *Ecc15* and a few other *Erwinia* spp. As these phytopathogens often use insects as vectors, one hypothesis for the selective benefit of acquiring *evf* is that this gene might be important for favoring bacterial transmission by strengthening the interaction of *Ecc15* with *Drosophila*. This hypothesis is supported by our results showing that the presence of *evf* allows the *Ecc15* strain to have higher loads at the initial stage of *Drosophila* larval infection. However, the rate of *Ecc15* elimination postinfection was the same in the WT and an *evf* mutant. This suggests that *evf* is promoting transmission of *Ecc15* by increasing the overall number of bacteria that reach the gut. Moreover, we show that larvae infected with *Ecc15* are developmentally delayed compared to noninfected larvae. This delay in larval development is most probably deleterious in an ecological scenario with strong competition between larvae and has been observed also in infection with Pseudomonas fluorescens (65). Although this delay is *evf* dependent, we do not know its molecular mechanism. Our results are, however, in agreement with previous reports showing that larvae infected with WT *Ecc15* had epithelial cell damage (7, 16, 17) and were smaller due to inhibition of gut proteolytic activity in turn promoted by *Drosophila*-associated *Lactobacillus* species (58). These studies, together with our results, show that *evf* expression in *Ecc15* has an overall deleterious effect on the host and thus that acquisition of *evf*, which enables higher host loads and is presumably beneficial for bacterial transmission, seems to have resulted in a trade-off for host fitness. Interestingly, *evf* homologues with low (below 40%) amino acid sequence identity, but with a predicted secondary structure highly similar to that of the Evf (66), can be found in other multihost bacteria such as the insect pathogen Photorhabdus luminescens (19) (locus PLU2433). P. luminescens colonizes the gut of Heterorhabditis bacteriophora, a nematode preying on insects (67, 68). The nematode enters through the insect’s respiratory and/or digestive tract and regurgitates the bacteria into its hemolymph. Once in the hemolymph, P. luminescens produces a battery of toxins that kill the insect, allowing the nematode to feed on the corpse and favoring P. luminescens recolonization (69–71). However, the role of this *evf* homologue in the establishment of interactions with either of P. luminescens hosts was never addressed.

Quorum sensing regulation is associated with tight control of density-dependent activation of genes encoding functions that are often essential for the establishment of host-microbe interactions (27). For instance, in the interaction between the squid Euprymna scolopes and V. fischeri, mutants with mutations in the quorum sensing system are less efficient in persisting in the light organ, being outcompeted by other strains (72, 73). Here, we show that *Ecc15* relies on quorum sensing to regulate both PCWDE and the *evf*-mediated developmental delay in infected *Drosophila* larvae. Moreover, overexpression of *evf* leads to a complete developmental arrest of larvae, eventually killing them. Therefore, one possible benefit of having *evf* expression under the control of these networks might be minimization of the detrimental effect that the *evf*-dependent infection has on the insect host while still enabling a transient infection. On the other hand, insects are attracted to rotten plant tissue, and if *evf* is important for promoting the interaction of *Ecc15* with its insect vector (*Drosophila*), synchronization of the expression of *evf* with that of the PCWDE might have been selected for as advantageous for bacterial dissemination. Indeed, we observed that *evf* and *pelA* (one of the PCWDE genes) are coexpressed during plant infection, even though *evf* is not required for plant maceration. Interestingly, although the quorum sensing system was necessary for expression of these genes and for maceration, lack of quorum sensing and of these traits did not affect bacterial loads during plant infection. One possibility is that maceration of the plant tissue is not necessary to sustain bacterial growth in the potato but might be needed to attract the insect and ensure transmission of bacteria to the next plant host. Therefore, it is possible that control of PCWDE expression and control of *evf* expression are intertwined such that, following colonization of the plant, *evf* expression is triggered, anticipating the appearance of the insect vector which is attracted to rotten plant tissue and thus maximizing the probability of establishing the interaction with this host vector. This phenomenon, called predictive behavior, is particularly common in symbiotic relationships where the microbe often experiences predictable cyclic environments (74). In mammalian hosts, a very predictable change that occurs during the transition from the outside environment to the oral cavity is an immediate increase in temperature followed by a decrease in the oxygen level. This phenomenon has been described for Escherichia coli gut colonization, where, coupled to an increase in temperature, downregulation of genes related to aerobic respiration is observed (75). Yersinia pestis, the etiological agent of plague, also experiences rapid environmental changes when transitioning between its two hosts. This bacterium colonizes the gut of fleas, particularly the proventriculus, forming a biofilm and blocking the digestive tract. This blockage induces a feeding behavior characterized by repetitive biting that increases the chances of successful transmission of *Yersinia* to the mammal host (76, 77). In the flea gut, *Yersinia* upregulates the expression of genes which do not provide an advantage in the colonization of the flea gut but which promote an increased resistance to phagocytosis by mammalian macrophages (78). This preconditioning mechanism, akin to a predictive behavior, provides Y. pestis with the ability to resist the first encounter with the host defenses, allowing subsequent expression of the remaining traits necessary to overcome the host immune system (79–81). Quorum sensing may play a role in activating the anticipatory genes in this bacterium because it has been shown to regulate genes important for colonization of flea and mammals (82, 83). Additionally, quorum sensing regulation in this bacterium is altered by changes in the metabolic environment related to different stages of the infection (84). Interestingly, P. luminescens also relies on different sets of genes to interact with each of its hosts (85, 86). This bacterium possesses two phenotypic variants: a primary one that is virulent to the insect and able to colonize the nematode and a secondary one that, while still virulent to the insect, is unable to support the development of the nematode (87, 88). However, it is not known if the traits involved in these processes are coregulated or not, but it is known that quorum sensing is required for the regulation of genes important in the interactions with the two hosts and thus that these genes might also be coregulated (89–92). Therefore, P. luminescens *and* Y. pestis are examples of bacteria which rely on different sets of virulence factors for the interactions with the different hosts and where quorum sensing plays a major role in regulating these traits. It is possible that anticipatory expression of virulence factors for the next stage may be regulated by quorum sensing in these bacteria, and in others that rely on multihost infections, as we have shown here for *evf* in *Ecc15*.

Our results show that, in *Ecc15*, the regulatory networks responding to self-produced quorum sensing signals and physiological cues sensed by the GAC system are used to coregulate expression of traits required to infect different hosts. Thus, the signal transduction mechanisms are the same even though the functions involved in the interactions with each plant or insect host are largely different. Therefore, our findings reinforce the idea of a central role of quorum sensing in the regulatory circuitry controlling the array of traits used by bacteria to interact with diverse hosts.

## MATERIALS AND METHODS

### Bacterial strains, plasmids, and culture conditions.

The strains and plasmids used in this study are listed in Table S1 of the supplemental material. All bacterial strains used were derived from the wild-type (WT) *Ecc15* strain (7). *Ecc15* and mutants were grown at 30°C with aeration in Luria-Bertani medium (LB). When specified, medium was supplemented with 0.4% polygalacturonic acid (PGA; Sigma catalog no. P3850) to induce expression of PCWDE. Escherichia coli DH5α was used for cloning procedures and was grown at 37°C with aeration in LB. When required, antibiotics were used at the following concentrations (mg liter^−1^): ampicillin (Amp), 100; kanamycin (Kan), 50; spectinomycin (Spec), 50; chloramphenicol (Cm), 25. To assess bacterial growth, optical density at 600 nm (OD_600_) was determined in a Thermo Spectronic Helios delta spectrophotometer.

10.1128/mBio.01292-20.9 TABLE S1 Strains and plasmids used in this study. Download Table S1, DOCX file, 0.02 MB. Copyright © 2020 Vieira et al. 2020 Vieira et al. This content is distributed under the terms of the Creative Commons Attribution 4.0 International license.

### Genetic and molecular techniques.

All primer sequences used in this study are listed in Table S2 in supplemental material. The *Ecc15* deletion mutants listed in Table S1 were constructed by chromosomal gene replacement with an antibiotic marker using the λ-Red recombinase system (93). Plasmid pLIPS, able to replicate in *Ecc15* and carrying the arabinose-inducible λ-Red recombinase system, was used (35). Briefly, the DNA region of the target gene, including approximately 500 bp upstream and downstream from the gene, was amplified by PCR and cloned into pUC18 (94) using restriction enzymes. These constructs, containing the target gene and its flanking regions, were divergently amplified by PCR, to introduce an XhoI restriction site in the 5′ and 3′ regions and to remove the native coding sequence of the target gene. The kanamycin cassette from pkD4 was amplified with primers also containing the XhoI restriction site. The fragment containing the kanamycin cassette was then digested with XhoI and was introduced into the XhoI-digested PCR fragment carrying the flanking regions of the target gene. The final construct, containing the kanamycin cassette flanked by the upstream and downstream regions of the target gene, was then amplified by PCR, and approximately 2 μg of DNA was electroporated into the parental strain (FDV31) expressing the λ-Red recombinase system from pLIPS, to favor recombination. To construct the plasmid carrying the *evf* promoter fused to GFP (pFDV54), a fragment of 503 bp containing the *evf* promoter was amplified from WT *Ecc15* DNA with the primers P1194 and P1195. This fragment was then digested with HindIII and SphI and ligated to pUC18. GFP was amplified from the pCMW1 (95) vector using primers P0576 and P0665. Both the GFP and pUC18-P_evf_ were digested with SphI and BamHI followed by ligation, and a 2-μl volume of the ligation reaction mixture was used to transform Dh5α (pFDV54). The same procedure was used for the P*_hor_*::*gfp* fusion using primers P1351 and P1352 for promoter amplification (493 bp) and primers P1353 and P1354 for GFP amplification. Digestions were made with enzymes HindIII/PstI and PstI/XbaI (pFDV84). For P*_pelA_*, primers P1941 and P1942 were used for promoter amplification (300 bp) and GFP was amplified using P1333 and P1334. Digestions were made using HindIII/XbaI and XbaI/SacI. For *hor* overexpression and for *expR1*, *expR2*, and *gacA* complementation, a NcoI site was introduced in pOM1-P*_evf_*::*gfp* with primers P1309 and P1310. *hor* was amplified using primers P1311 and P1312 from WT template DNA. Then, both the plasmid and the fragment carrying *hor* were digested with NcoI and SacI and subsequently ligated (pFDV104). *expR1* and *gacA* were amplified using primers P1943 and P1944 and primers P1947 and P1948 from WT template DNA. Then, both the plasmid and the fragment carrying the desired gene were digested with EcoRI and SacI and subsequently ligated (pFDV104). For *expR2* complementation, a fragment was amplified from WT template DNA with primers P1958 and P1959, digested with XmnI, and ligated into the plasmid containing the *expR1* and P*_evf_*::gfp expression reporter. pOM1-mCherry was constructed by digesting pOM1 with XmnI and ligating a fragment of 825 bp amplified with primers P1789 and P1790 from genomic DNA of strain RB290 containing the constitutive mCherry fusion.

10.1128/mBio.01292-20.10 TABLE S2 Primers used in this study. Download Table S2, DOCX file, 0.01 MB. Copyright © 2020 Vieira et al. 2020 Vieira et al. This content is distributed under the terms of the Creative Commons Attribution 4.0 International license.

PCR for cloning purposes was performed using the proofreading enzyme Bio-X-ACT (Bioline). Other PCRs were performed using Dream *Taq* polymerase (Fermentas). Digestions were performed with Fast Digest enzymes (Fermentas), and ligations were performed with T4 DNA ligase (New England Biolabs). All cloning steps were performed in either E. coli DH5α or WT *Ecc15*. All mutants and constructs were confirmed by PCR amplification and subsequent Sanger sequencing performed at the Instituto Gulbenkian de Ciência sequencing facility.

### Pectate lyase activity assay.

*Ecc15* and mutants were grown overnight in LB with 0.4% PGA, inoculated into fresh media to a starting OD_600_ of 0.05, and incubated at 30°C with aeration. After 6 h of incubation, aliquots were collected to evaluate growth and to analyze pectate lyase (Pel) activity in cell-free supernatants, using the previously described procedure (59) based on the thiobarbituric acid colorimetric method (96). Each experiment included at least 5 independent cultures per genotype and was repeated on 3 independent days.

### Plant virulence assay.

Plant virulence was analyzed by assessing the maceration of potato tubers with a protocol adapted from previous studies (35, 97). Potatoes were washed and surface sterilized by soaking for 10 min in 10% bleach, followed by 10 min in 70% ethanol. Overnight cultures in LB broth were washed twice and diluted to an OD_600_ of 0.05 in phosphate-buffered saline (PBS). Thirty-microliter aliquots were then used to inoculate the previously punctured potatoes. Potato tubers were incubated at 28°C at a relative humidity above 90% for 6, 24, or 48 h. After incubation, potatoes were sliced, and macerated tissue was collected and weighed.

### Promoter expression assays.

*Ecc15* bacterial cells carrying the different plasmid-borne promoter reporter fusions were grown overnight in LB supplemented with spectinomycin (LB + Spec), inoculated into fresh medium at a starting OD_600_ of 0.05, and incubated at 30°C with aeration. At the indicated time points, aliquots were collected to assess growth and the expression of the reporter fusion. For the analyses of reporter expression, aliquots of the cultures were diluted 1:100 in PBS and expression was measured by flow cytometry (LSRFortessa; BD) and analyzed with Flowing Software v 2.5.1, as previously described (59). A minimum of 10,000 green fluorescent protein (GFP)-positive single cells were acquired per sample. Expression of the promoter-*gfp* fusions is reported as the median GFP expression of GFP-positive single cells in arbitrary units. Each experiment included at least 5 independent cultures per genotype and was repeated on 3 independent days.

### *Drosophila* stocks.

DrosDel *w^1118^* isogenic stock (*w^1118^ iso*) was used in all experiments (98, 99). Stocks were maintained at 25°C in standard corn meal fly medium composed of water (1.1 liter), 45 g molasses, 75 g of sugar, 10 g agar, 70 g cornmeal, and 20 g yeast. Food was autoclaved and cooled to 45°C before addition of 30 ml of a solution containing 0.2 g of carbendazim (Sigma), 100 g of methylparaben (Sigma), and 1 liter of absolute ethanol. Experiments were performed at 28°C

### Developmental delay and bacterial CFU assays.

Egg-laying took place in cages containing adult flies at a ratio of 3 females to 1 male. To synchronize the embryo stages, flies were initially incubated for 1 h at 25°C to lay prior fertilized eggs. After this initial incubation, flies were transferred to new cages where eggs were laid for 4 to 6 h in the presence of standard corn meal fly medium. After this period, eggs were removed and incubated at 25°C for 72 h to obtain L3-stage larvae. For bacterial infections, third-instar larvae were placed in a 2-ml Eppendorf tube containing 200 μl of concentrated bacterium pellet (OD_600_ = 200) from an overnight culture and 400 μl of standard corn meal fly medium. Larvae, bacteria, and food were then thoroughly mixed using a spoon, and the Eppendorf tube was closed with a foam plug and incubated at room temperature (RT) for 30 min. The mix was then transferred to a 25-ml plastic tube containing 7.5 ml of standard corn meal fly medium and incubated at 28°C. To assess development of the larvae postinfection, pupa were counted every 12 h for 5 days. For CFU counts, larvae were inoculated as described above. At each time point, 5 larvae were randomly collected, surface sterilized for 10 s in ethanol 70%, and washed with Milli-Q water. Individual larvae were then transferred to Eppendorf tubes containing 300 μl of 1× PBS and homogenized with a blender. The homogenate was diluted 100-fold, and serial dilutions were plated in LB. Plates were incubated overnight at 30°C.

### Promoter expression assays in plant infections.

*Ecc15* bacteria carrying either of the different plasmid-borne promoter GFP reporter fusions (P*_evf_*::*gfp* or P*_pelA_*::*gfp*) and a constitutive mCherry fusion were grown overnight in LB supplemented with spectinomycin (LB + Spec). Bacterial cells (2 ml) were collected and washed twice in 1× PBS. Potatoes were infected as described in the plant virulence assay section. At the indicated time points, potatoes were sliced, and macerated tissue was weighted. To isolate bacterial cells from potato tissue, approximately 0.5 g (or all the soft tissue if the weight was lower than 0.5 g) was introduced into an Eppendorf tube containing 250 μl of 1× PBS. Bacterial cells were isolated from the plant tissue by adapting the previously described protocol (100). Briefly, tissue was mechanically disrupted using a pipette tip before addition of 750 μl of 1× PBS to the collection tube. The disrupted pellets were then subjected to 4 iterations of mixing using a vortex mixer for 15 s at medium speed followed by centrifugation at 2,000 rpm at RT for 1 min, recovery of the 750 μl of 1× PBS into a new tube, and replacement of that volume of PBS before the next iteration. The resulting 3 ml of isolated cells was pelleted for 5 min at 4,000 × *g* for 5 min at RT, the supernatant was discarded, and the cells were resuspended in 1 ml of 1× PBS. For CFU counts at the infection site, 100 μl of the recovered cells was diluted, plated in LB + Spec, and incubated overnight at 30°C and red colonies were counted. For the analyses of reporter expression, aliquots of the recovered cells were diluted 1:100 in PBS and expression was measured by flow cytometry (LSRFortessa; BD) and analyzed with Flowing Software v 2.5.1, as previously described (59). Potatoes were also infected with an *Ecc15* strain carrying a plasmid with a constitutive mCherry promoter fusion and no GFP fusion; bacteria collected from these infected potatoes were used to define the gating that allows distinguishing between potato debris and bacterial cells. A minimum of 10,000 GFP and mCherry doubly positive single cells were acquired per sample. Expression of the promoter-*gfp* fusions is reported as the median level GFP expression of doubly fluorescence-positive single cells in arbitrary units. Each experiment included at least 5 independent cultures per genotype, and all experiments were repeated on 2 independent days.

### Statistical analysis.

Statistical analyses were performed in R (101), and graphs were generated using the package ggplot2 (102). All experiments were analyzed using linear mixed-effect models (package lme4, updated version 1.1-20 [103]). Significance of interactions between factors was tested by comparing models fitting the data with and without the interactions using analysis of variance (ANOVA). Models were simplified when interaction data were not statistically significant. Multiple comparisons of the estimates from fitted models were performed with a Tukey HSD (honestly significant difference) test (packages lmerTest [104] and multicomp [105, 106]). A letter is assigned to each statistical group; differing letters stand for statistically significant differences.
