# Supplementary material for: Erwinia carotovora Quorum Sensing System Regulates Host-Specific Virulence Factors and Development Delay in Drosophila melanogaster
Source: mBio. 2020 Jun 23;11(3):e01292-20. doi: 10.1128/mBio.01292-20 (PMC7315124; doi:10.1128/mBio.01292-20)
Supplement: TABLE S1 [file mBio.01292-20-st001.docx]

**Table S1.** Strains and plasmids used in this study

| **Strain** | **Parental strain** | | **Relevant Genotype** | **Source** | |
| --- | --- | --- | --- | --- | --- |
| ***E. carotovora*** | | | | | |
| ***Ecc15*** |  | | Wild-type (WT) | (16) | |
| **FDV31** | *Ecc15* | | WT carrying pLIPS | (35) | |
| **FDV51** | *Ecc15* | | *expI::cm* | This study | |
| **FDV42** | *Ecc15* | | *gacA::kan* | This study | |
| **FDV163** | *Ecc15* | | *expIexpR1::cm*/*expR2::kan* | This study | |
| **FDV22** | *Ecc15* | | *hor::kan* | This study | |
| **FDV54** | *Ecc15* | | WT carrying pFDV54 | This study | |
| **FDV56** | FDV51 | | *expI::cm* carrying pFDV54 | This study | |
| **FDV58** | FDV42 | | *gacA::kan* carrying pFDV54 | This study | |
| **FDV165** | FDV163 | | *expIexpR1::cm*/*expR2::kan* carrying pFDV54 | This study | |
| **FDV60** | FDV22 | | *hor::kan* carrying pFDV54 | This study | |
| **FDV84** | *Ecc15* | | WT carrying pFDV84 | This study | |
| **FDV92** | FDV51 | | *expI::cm* carrying pFDV84 | This study | |
| **FDV86** | FDV42 | | *gacA::kan* carrying pFDV84 | This study | |
| **FDV104** | *Ecc15* | | WT carrying pFDV104 | This study | |
| **FDV114** | FDV51 | | *expI::cm* carrying pFDV104 | This study | |
| **FDV127** | FDV42 | | *gacA::kan* carrying pFDV104 | This study | |
| **FDV517** | FDV42 | | *gacA::kan* carrying pFDV502 | This study | |
| **FDV519** | FDV163 | | *expIexpR1*::*cm*/*expR2*::*kan* carrying pFDV519 | This study | |
| **FDV407** | *Ecc15* | | WT carrying pFDV407 | This study | |
| **FDV418** | FDV51 | | *expI*::*cm* carrying pFDV407 | This study | |
| **FDV200** | *Ecc15* | | WT carrying pFDV200 | This study | |
| **FDV505** | FDV51 | | *expI*::*cm* carrying pFDV200 | This study | |
| **FDV519** | *Ecc15* | | WT carrying pFDV519 |  | |
| ***E.coli*** | | | | | |
| RB290 | K-12 MG1655 | | WT | Provided by Roberto Balbontin  (106) | |
|  |  | |  |  | |
| **Plasmids** |  | **Description** | | | **Source** |
| **pOM1** |  | Cloning vector, *Spec^r^* | | |  |
| **pUC18** |  | Cloning vector, *Amp^r^* | | |  |
| **pLIPS** |  | pOM1 vector containing λ red recombinase system, *Spec^r^* | | | (35) |
| **pFDV54** |  | pOM1 vector containing promoter *evf::gfp*, *Spec^r^* | | | This study |
| **pFDV104** |  | pOM1 vector containing a promoter lac::*hor* and a promoter *evf::gfp* | | | This study |
| **pFDV84** |  | pOM1 vector containing a promoter *hor::gfp*, *Spec^r^* | | | This study |
| **pFDV502** |  | pOM1 vector containing a promoter *evf*::*gfp* and a *gacA* gene | | | This study |
| **pFDV519** |  | pOM1 vector containing a promoter *evf*::*gfp,* a *expR1* and a *expR2 gene* | | | This study |
| **pFDV407** |  | pOM1 vector containing a promoter *pelA*::*gfp* and a constitutive P_tet_::*mCherry* | | | This study |
| **pFDV200** |  | pOM1 vector containing a promoter *evf*::*gfp* and a constitutive P_tet_::*mCherry* | | | This study |
| **pFDV567** |  | pOM1 vector containing a constitutive Ptet::*mCherry* | | | This study |
