# Supplementary material for: Erwinia carotovora Quorum Sensing System Regulates Host-Specific Virulence Factors and Development Delay in Drosophila melanogaster
Source: mBio. 2020 Jun 23;11(3):e01292-20. doi: 10.1128/mBio.01292-20 (PMC7315124; doi:10.1128/mBio.01292-20)
Supplement: TABLE S2 [file mBio.01292-20-st002.docx]

**Table S2.** Primers used in this study

| **Primer Name** | **Sequence** |
| --- | --- |
| 1108-Redsystem(pKD46)FWsphI | CCTTACGCATGCCATCGATTTATTATGACAA |
| 1109-Redsystem(pKD46)RVXbaI | CGAGCTTCTAGATACCCATGGATTCTTCGTCT |
| 1127-500Hor500RVSalI | CGAGCTGTCGACGCTAAACAGGTGCAGACCGT |
| 1128-500Hor500FWSalI | CCTTACGTCGACTCAATAAATAGAGTTGTCGCGGG |
| 1130-500gacA500FwSalI | CCTTACGTCGACTATGATGTTCACTATGGACG |
| 1131-500gacA500RvSalI | CGAGCTGTCGACGATATTGCAGGCAGGGGCG |
| 1087-HorDelRVXhoI | CGAGCTCTCGAGCACCTCTCCTTATTGTTAGC |
| 1088-HorDelFWXhoI | CCTTACCTCGAGCTAAATTTGGGTTACGCAGA |
| 1132-DelGacARvXhoI | CGAGCTCTCGAGGAATAATTCTCCAAAAAAGGG |
| 1133-DelGacAFwXhoI | CCTTACCTCGAGGAGTTTCGATGCGTCGGCAT |
| 1134-DelExpIFwXhoI | CCTTACCTCGACTTGCACAGGCTTGATGAGCTGTA |
| 1135-DelExpIRvXhoI | CGAGCTCTCGAGCCTCCATTGAAAAGTTAATAC |
| 1136-500ExpI500FwSalI | CCTTACGTCGACGAATACCGTGTCTGACAACC |
| 1137-500ExpI500RvSalI | CGAGCTGTCGACATCGCCTTTCTCTTGGGAGA |
| 1186-HorDelFw | AATCGTCAGTTATTACAATGGT |
| 1187-HorDelRv | TATGATGAAGCGTTTGCTTGTG |
| 1190-ExpIDelFw | TCAGGCGCTGATGCTGCGTGAT |
| 1191-ExpIDelRv | TCCAGTTATCCCGATGAATGGG |
| 1192-GacADelFw | GGGCGTTACCGCTGACGCGACA |
| 1193-GacADelRV | CAGGCGAACATAGTCAACCTGC |
| 1309-NcoIsiteFW | CCTTACCCATGGTTACGAATTCGAGCT |
| 1310-NcoIsiteRV | CCTTACCCATGGTCATAGCTGTTTCCT |
| 1311-horNcoIFW | CCTTACCCATGGAATTGCCATTAGGAT |
| 1312-horSacIRV | CCTTACGAGCTCCTACGCTTGATTTTCATG |
| 1351-pHor(500bp)_FW | CCTTACAAGCTTTAGAGTTGTCGCAGGAGGTG |
| 1352-pHor(500bp)_RV | CCTTACCTGCAGCACCTCTCCTTATTGTTAGC |
| 1194-pEvfFw | CCTTACAAGCTTTGCTTACAGGAAACCAACAA |
| 1195-pEvf_Rv | CGAGCTGCATGCAATCACTCCTATTGTGGTGG |
| 1411-500evf500FwSalI | CCTTACGTCGACTGCTTACAGGAAACCAACAA |
| 1412-500evf500RvSalI | CGAGCTGTCGACGCATTACTCTACACTTTTCTGAC |
| 1413-EvfDelXhoIFw | CCTTACCTCGAGTTCATAAAATATAGTCAGGG |
| 1414-EvfDelXhoIRv | CGAGCTCTCGAGAATCACTCCTATTGTGGTGG |
| 1415-EvfDelConfFw | CGTTCCCGTTGAAGTCATGG |
| 1416-EvfDelConfRv | CTGGATCGCTGGCTCCAAAC |
| 1235-500-ExpR2-500SalIFw | CCTTACGTCGACGGAGAAGGACGGGAAAGGTA |
| 1236-500-ExpR2-500SalIRv | CGAGCTGTCGACTTGATGATTCGGTGCTGGCG |
| 1237-DelExpR2XhoIFw | CCTTACCTCGAGTGTCATCACGTCTATTTCACT |
| 1238-DelExpR2XhoIRv | CGAGCTCTCGAGGTAACGGCCTCAATAAAAAGCG |
| 1239-ExpR2DelConFw | CTAAAACATTAGCCTCACCGCCG |
| 1240-ExpR2DelConRv | CTAACATGGGCGCGTGTGTATCG |
| 1241-500-ExpR1-500SalIFw | CCTTACGTCGACCACGATTGACGCCAGCTATGA |
| 1242-500-ExpR1-500SalIRv | CGAGCTGTCGACGGCATCAAAGATAACACCGT |
| 1243-DelExpR1XhoIFw | CCTTACCTCGAGAGTTACAGCTCATCAAGCCT |
| 1244-DelExpR1XhoIRv | CGAGCTCTCGAGCCTCAGTCTGAAGAATCAAC |
| 1245-ExpR1DelConFw | CGCCTGGGATCAGGGAGCAA |
| 1246-ExpR1DelConRv | GAAACGAAATCAGAAGAGCT |
| 1353-GFP(noRBS)_FW | CCTTACCTGCAGATGGCTAGCAAAGGAGAAGAACTCT |
| 1354-GFP(noRBS)_RV | CCTTACTCTAGAACCGGATCCTCAGTTGTACAGTTCA |
| 0665-GFP(noRBS)_RV | CCTTACGGATCCTCAGTTGTACAGTTCATCCATGCCA |
| 0576-GFP(noRBS)_FW | CCTTACGCATGCATGGCTAGCAAAGGAGAAGAACTCT |
| 1333-GFP_FW_XbaI | CGTTCTAGAATGGCTAGCAAAGGAGAAGAACTC |
| 1334-GFP_RV_SacI | GTCGAGCTCGTAAACCGGATCCTCAGTTGTACAGT |
| 0531_pOM1seq_R | ATTAAGTTGGGTAACGCCAGGGTTTTCCCAGTC |
| 0752-pOM1_seq2_F | CGCCCAATACGCAAACCGCCTCTCCCCGCGCGT |
| 0782- pKD3/4 XhoI Fw | AGTCTCGAGTTGTGTAGGCTGGAGCTGCTTC |
| 0783- pKD3/4 XhoI Rv | GCGCTCGAGCCATATGAATATCCTCCTTAG |
| 1943-expR1comp-EcoR1-FW | CCTGAATTCCGCAATCTCGACGATGTG |
| 1944-expR1comp-sacI-RV | CCTGAATTCCGCAATCTCGACGATGTG |
| 1947-gacAcomp-EcoR1-FW | CCTGAATTCTATGATGTTCACTATGGACG |
| 1948-gacAcomp-sacI-RV | CCTGAGCTCCTCTCACTCACTACTTAACAATG |
| 1958-expR2comp-XmnI-FW | CCTGAAGCAGTTCCACCCTAAATTCAGTAGGCAG |
| 1959-expR2-XmnI-RV | CCTGAAGCAGTTCGTGAAATAGACGTGATGACAA |
| 1941-PpelA-FW-hindIII | CTTGGTAAGCTTTTCTTCTCTTCACA |
| 1942-PpelA-RV-xbaI | CTTTCTAGAGTGTTTTTCCTTGTAAATTAAACG |
| 1789-RB290_mcherry_FW | CTTGAAGCAGTTCTCTTCACCTCGAGTCCCTAT |
| 1790-RB290_mcherry_RV | CTTGAAGCAGTTCTTACTTGTACAGCTCGTCCA |
